# miR-30d suppresses proliferation and invasiveness of pancreatic cancer by targeting the SOX4/PI3K-AKT axis and predicts poor outcome

**DOI:** 10.1038/s41419-021-03576-0

**Published:** 2021-04-06

**Authors:** Xiaodong Xu, Ke Zong, Xinxing Wang, Dongwei Dou, Pengwei Lv, Zhe Zhang, Hongwen Li

**Affiliations:** 1 Department of Breast Surgery, The First Affiliated Hospital of Zhengzhou University, No. 1 Jianshe east Road, 450000 Zhengzhou, China; 2 Department of Hepatobiliary and Pancreatic Surgery, The First Affiliated Hospital of Zhengzhou University, No. 1 Jianshe east Road, 450000 Zhengzhou, China; 3 Department of Oncology, The First Affiliated Hospital of Zhengzhou University, No. 1 Jianshe east Road, 450000 Zhengzhou, China

**Keywords:** Oncogenes, Oncogenesis

## Abstract

Aberrant expression of miR-30d is associated with the development and progression of several human cancers. However, its biological roles and underlying mechanisms in pancreatic cancer are largely unknown. The expression of miR-30d in pancreatic cancer was evaluated in public databases and further valuated by real-time quantitative PCR, western blot, and immunohistochemistry in a cohort of pancreatic cancer patients. The role of miR-30d in the proliferation and metastasis of pancreatic cancer cells was determined using in vitro and in vivo assays. Bioinformatics analyses were performed to examine potential target genes of miR-30d. Luciferase reporter assay and functional rescue experiments were used to elucidate the mechanisms of miR-30d. miR-30d was found frequently decreased in pancreatic cancer compared with nontumor tissues, and downregulation of miR-30d predicted poor prognosis and early relapse of pancreatic cancer patients. Overexpression of miR-30d significantly repressed the growth and metastasis of pancreatic cancer cells both in vitro and in vivo. Bioinformatics analyses identified sex-determining region Y-box 4 (SOX4) as a target gene of miR-30d. Mechanically, miR-30d exerted its tumor suppressive effect by directly targeting SOX4, which caused inhibition of the PI3K-AKT signaling pathway. Overexpression of SOX4 partially antagonized the inhibitory effects of miR-30d. Our study demonstrated that dysregulation of the miR-30d/SOX4/PI3K-AKT axis promotes the development and progression of pancreatic cancer. These findings suggest miR-30d as a promising and reliable therapeutic target for pancreatic cancer.

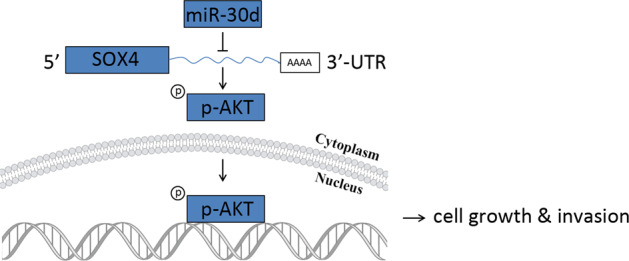

## Background

Pancreatic ductal adenocarcinoma (PDAC) is one of the most common malignancies worldwide and characterized by a high lethality rate^1^. Although progress has been made in the treatment of pancreatic cancer, the survival rate of pancreatic cancer is still very poor^2–4^. Therefore, better understanding of the underlying molecular mechanisms in PDAC with the aim of identifying novel therapeutic targets is critical.

MicroRNAs (miRNAs) are small non-coding RNAs that regulate mRNA expression by a post-transcriptional mechanism^5^. Accumulating studies have showed that miRNAs are key players in cancer pathogenesis through their functions in regulating cell motility, apoptosis, cell cycle, proliferation, and stress response in various cancers^6,7^. Several studies showed that miRNAs are of functional significance and might be promising prognostic biomarkers in pancreatic cancer. For example, miR-92b-3p suppresses pancreatic cancer formation by targeting Gabra3 both in vitro and in vivo^8^. miRNA-141 inhibits the growth and invasion of pancreatic cancer cells by targeting MAP4K4^9^. miR-1181 inhibits cancer stem cell-like characteristics of pancreatic cancer cells by suppressing both SOX2 and STAT3^10^.

Previous studies showed that miR-30d, a member of the miR-30 family, is dysregulated in multiple cancers and acts as an oncogene or tumor suppressor in different tumors. For instance, upregulation of miR-30d promotes invasion and migration of breast cancer cells by targeting KLF-11 and pSTAT3^11^. In contrast, miR-30d inhibits cell proliferation and motility in non-small cell lung cancer by directly targeting CCNE2^12^. miR-30d targets beclin-1 to impact the sensitivity of anaplastic thyroid carcinoma cells to cisplatin^13^. However, the expression and potential function of miR-30d in the development and progression of pancreatic cancer is still unknown.

SOX4 is a member of the highly conserved SOX family of transcription factors^14^, which play a vital role in embryonic development and cell fate determination^15–17^. Accumulating evidence indicates that SOX4 is an oncoprotein in numerous cancers including breast, liver, colorectal, and esophageal cancers^18–21^.

In the present study, we integrated public expression datasets and bioinformatics predictions and determined the expression and prognostic value of miR-30d in pancreatic cancer. Gain and loss of function experiments were performed to determine the biological roles of miR-30d. We found that miR-30d is downregulated in pancreatic cancer tissues and cell lines. Furthermore, miR-30d inhibited pancreatic cell growth and invasion by targeting SOX4 via inactivating the PI3K-AKTsignaling pathway. These findings demonstrated that dysregulation of the miR-30d–SOX4–PI3K-AKT axis is of great importance for the carcinogenesis of pancreatic cancer and might be a potential therapeutic target.

## Results

### miR-30d is downregulated in pancreatic cancer and predicts poor prognosis

**Fig. 1:**
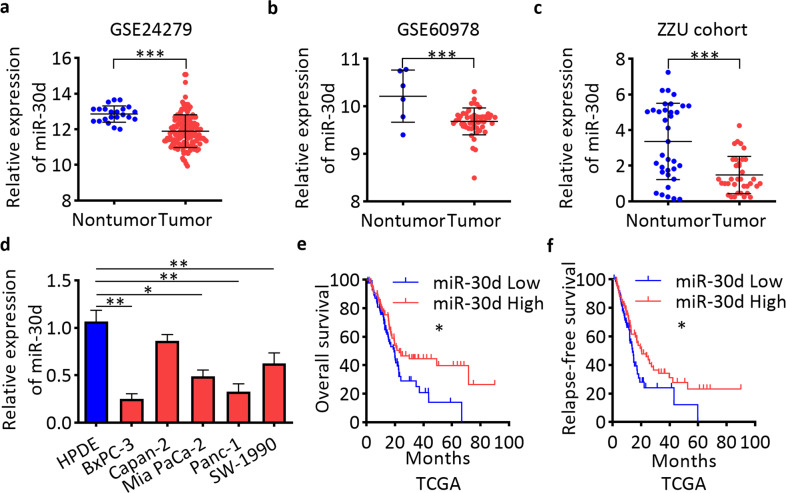
miR-30d is downregulated in pancreatic cancer. **a** Expression of miR-30d in the GSE24279 dataset (*n* = 136). **b** miR-30d expression in the GSE60978 dataset (*n* = 51). **c** miR-30d expression was detected in pancreatic cancer tissues compared with matched adjacent non-tumor tissues from the ZZU cohort by RT-qPCR analysis (*n* = 35). **d** miR-30d expression was examined in five pancreatic cancer cell lines and HPDE cells by RT-qPCR. U6 served as the internal reference for miR-30d. **e**, **f** Kaplan–Meier analysis of the OS (*n* = 171) and RFS (*n* = 132) of pancreatic cancer patients from the TCGA cohort. Patients were grouped according to median miR-30d expression. *P < 0.05, **P < 0.01, ***P < 0.001.

We mined public GEO datasets and found that miR-30d was significantly downregulated in pancreatic cancer compared with non-tumor tissues (Fig. 1a, b). We then examined the expression level of miR-30d in 35 pairs of pancreatic cancer tissues and adjacent non-tumor tissues from The First Hospital of Zhengzhou University (ZZU cohort) by RT-qPCR and found downregulation of miR-30d in pancreatic cancer tissues (Fig. 1c). We also examined the expression level of miR-30d in pancreatic cancer cell lines by RT-qPCR, and the results showed that miR-30d expression was lower in all five pancreatic cancer cell lines compared with the normal HPDE cell line (Fig. 1d). To study the clinical significance of miR-30d in pancreatic cancer, we mined the TCGA PAAD dataset and performed Kaplan–Meier analysis. Compared with patients in the low miR-30d expression group (*n* = 86), patients in the high miR-30d expression group (*n* = 85) showed longer overall survival (OS) (median: 23.06 months vs. 19.45 months; Fig. 1e) and longer relapse-free survival (RFS) (median: 20.27 months vs. 14.03 months; Fig. 1f). Univariate and multivariate Cox regression analyses showed that miR-30d was an independent prognostic factor for both prognosis and recurrence (Supplementary Tables 1 and 2). These results suggest that miR-30d is downregulated in pancreatic cancer tissues and cell lines and predicts poor prognosis and early recurrence in pancreatic cancer patients.

### miR-30d inhibits pancreatic cancer cell growth and invasion in vitro

**Fig. 2:**
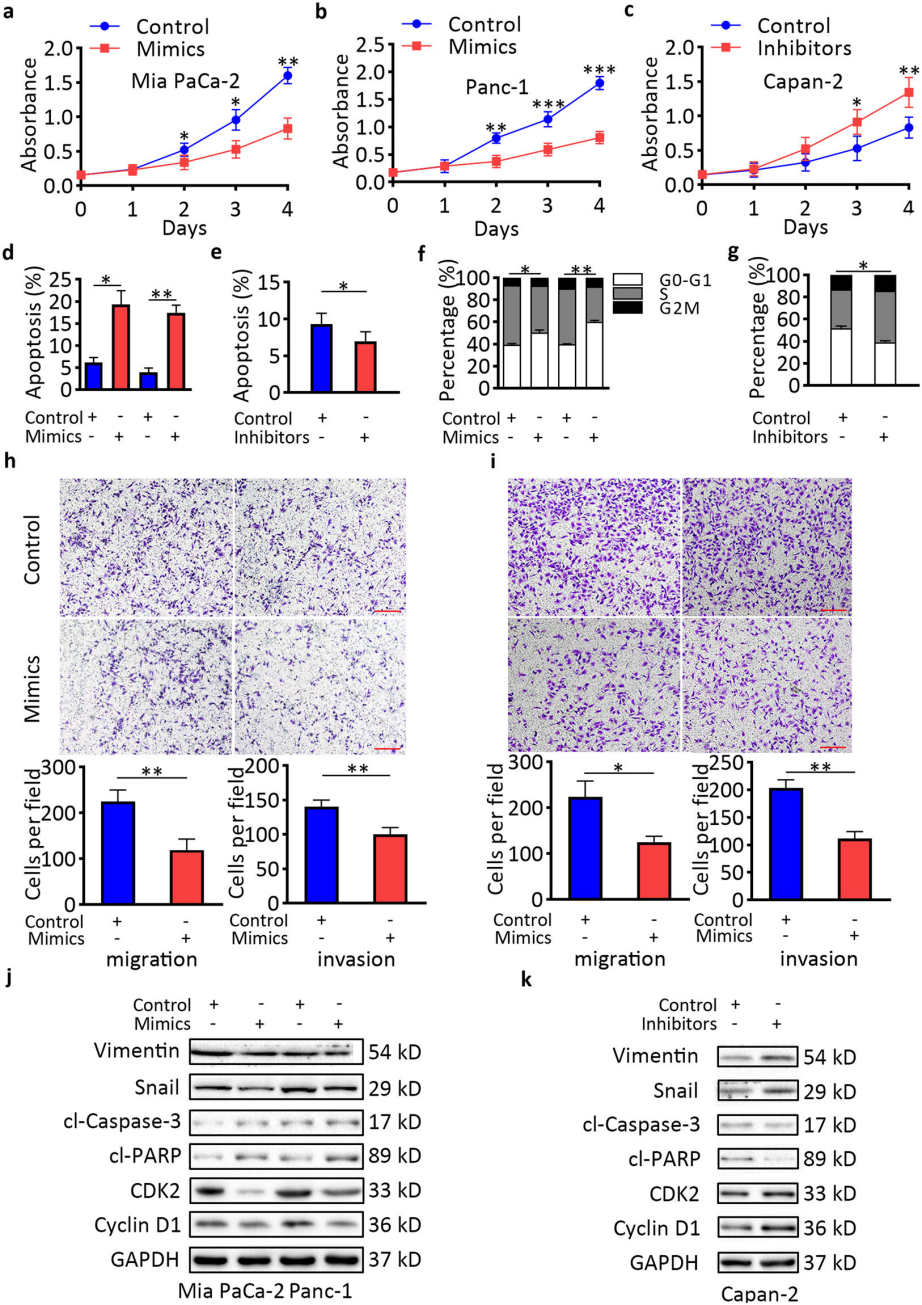
miR-30d inhibits pancreatic cancer cell growth and invasion in vitro. **a**–**c** CCK8 assays of pancreatic cancer cells after transient transfection with lipofectamine. **d** Cell apoptosis analysis by flow cytometry after transfection of miR-30d mimics in Mia PaCa-2 (left) and Panc-1 cells (right). **e** Cell apoptosis analysis by flow cytometry after transfection of miR-30d inhibitors in Capan-2 cells. **f** Cell cycle analysis by flow cytometry after transfection of miR-30d mimics in Mia PaCa-2 (left) and Panc-1 cells (right). **g** Cell cycle analysis by flow cytometry after transfection of miR-30d inhibitors in Capan-2 cells. **h**, **i** Transwell assays without or with matrigel coating to assess cell migration and invasion activities, respectively, in Mia PaCa-2 (left) and Panc-1 cells (right). Scale bar, 50 μm (red line). **j**, **k** Western blot analysis of key cell apoptosis, cell cycle, and cell invasion markers. *P < 0.05, **P < 0.01, ***P < 0.001.

Given the findings that downregulation of miR-30d was associated with a worse outcome in pancreatic cancer patients, we hypothesized that miR-30d might function as a tumor suppressor in pancreatic cancer. We investigated the role of miR-30d in pancreatic cancer by transfecting miR-30d mimics or control sequences into Mia PaCa-2 and Panc-1 cells; these have low endogenous expression of miR-30d, and knocked down miR-30d expression by transfection of miR-30d inhibitors or control sequences into Capan-2 cells, which have relatively high endogenous expression of miR-30d. Knockdown and overexpression of miR-30d were confirmed by RT-qPCR (Supplementary Fig. S1). CCK-8 and colony formation assays showed that re-expression of miR-30d significantly inhibited the proliferation of both Mia PaCa-2 and Panc-1 cells compared with the control group (Fig. 2a, b and Supplementary Fig. S2). Conversely, knockdown of miR-30d expression promoted the growth of Capan-2 cells compared with the control group (Fig. 2c and Supplementary Fig. S2). Flow cytometry was used to investigate the effect of miR-30d on cell apoptosis and cell cycle progression. Compared with the control group, cells overexpressing miR-30d showed an increased proportion of cell apoptosis (Fig. 2d) and cell cycle arrest at G1/S phase (Fig. 2f and Supplementary Fig. S3). In Capan-2 cells, inhibition of miR-30d expression reduced the apoptosis and promoted cell cycle progression (Fig. 2e, g and Supplementary Fig. S3). Since our previous data suggested that downregulation of miR-30d predicted early recurrence of pancreatic cancer patients, we next studied the influence of miR-30d on PDAC cell motility and invasiveness by transwell assays. Both the migratory and invasive capacities of Mia PaCa-2 and Panc-1 cells were suppressed after transfection of miR-30d mimics compared with controls (Fig. 2h, i). Conversely, inhibition of miR-30d promoted the migration and invasion of Mia PaCa-2 cells (Supplementary Fig. S4). Changes of cell apoptosis, cell cycle, and cell invasion were also confirmed by western blotting analysis of the key regulators in these processes (Fig. 2j, k). Taken together, these results indicate that miR-30d suppresses the proliferation and invasion of pancreatic cancer cells in vitro.

### SOX4 is a direct target of miR-30d

**Fig. 3:**
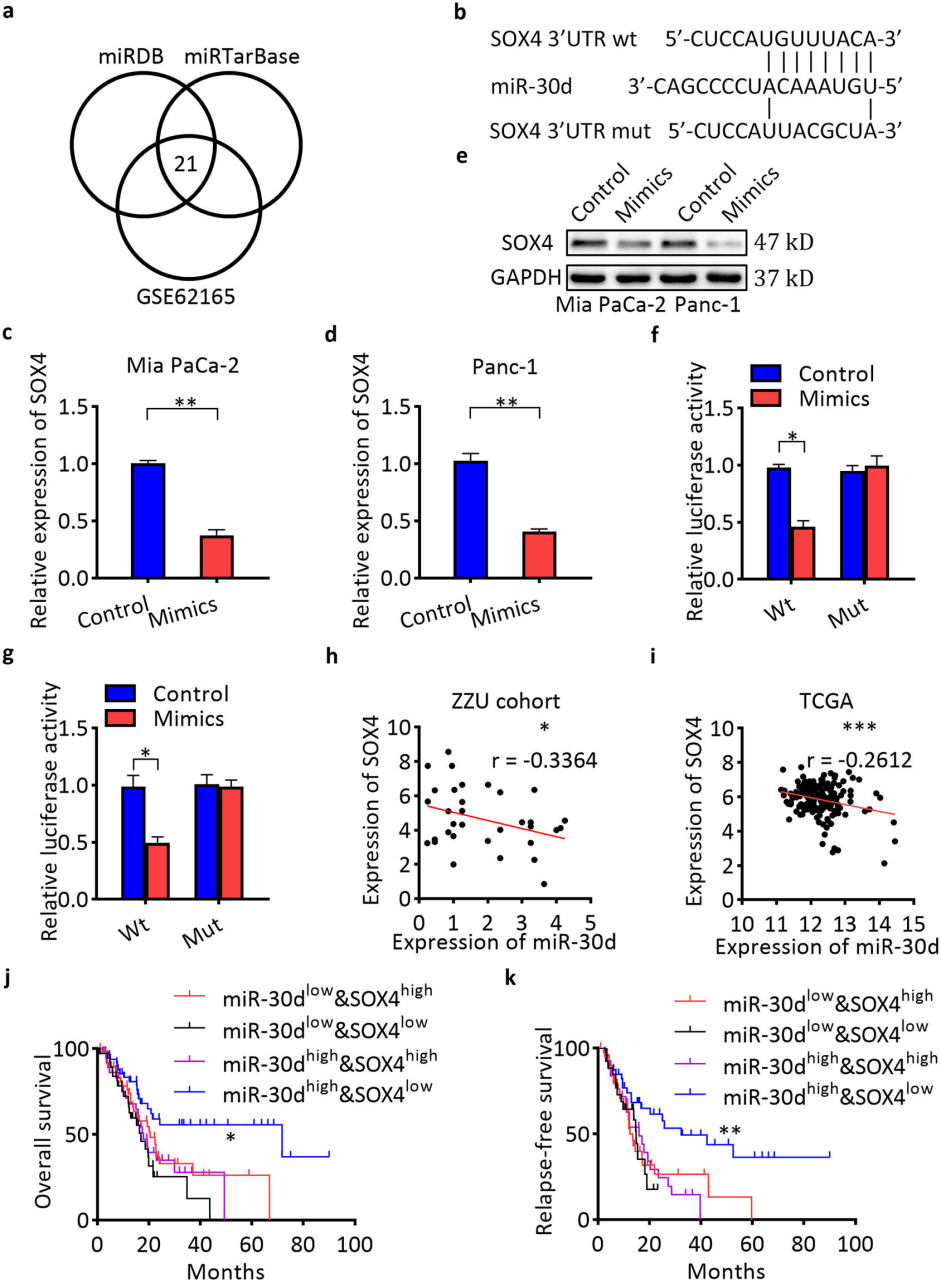
SOX4 is a direct target of miR-30d. **a** Venn diagram analysis to determine potential miR-30d targets. **b** Predicted binding sequences between miR-30d and the 3′-UTR of SOX4. **c**–**e** Levels of SOX4 mRNA and protein in pancreatic cancer cells after transient transfection with lipofectamine by RT-qPCR and western blot, respectively. **f**, **g** Relative luciferase activity in Mia PaCa-2 and Panc-1 cells after transient transfection with lipofectamine. **h** Correlations between miR-30d and SOX4 mRNA expression in pancreatic cancer tissues (*n* = 35). **i** Correlations between miR-30d and SOX4 mRNA expression in patients in the TCGA dataset (*n* = 171). **j** OS status analysis of pancreatic cancer patients in TCGA based on SOX4 and miR-30d expression (*n* = 171). **k** RFS status analysis of pancreatic cancer patients in TCGA based on SOX4 and miR-30d expression (*n* = 131). *P < 0.05, **P < 0.01, ***P < 0.001.

To identify potential target genes of miR-30d, bioinformatics analyses were performed using two algorithms of miRDB and miRTarBase (Fig. 3a). The predicted target gene should also be upregulated in GSE62165. Among the 21 candidate targets (Supplementary Table 3), we selected SOX4 as one of the likely candidates. We identified a putative miR-30d binding site in its 3′-untranslated region (UTR) (Fig. 3b). Overexpression of miR-30d resulted in dramatic downregulation of SOX4 mRNA and protein levels in both cells (Fig. 3c–e). To verify whether SOX4 is directly targeted by miR-30d, a dual-luciferase reporter assay was performed using a luciferase reporter vector containing the 3′-UTR of SOX4 mRNA. miR-30d mimics significantly downregulated the luciferase activity of the reporter compared with controls (Fig. 3f, g). However, no effect was observed on luciferase activity when miR-30d mimic was transfected with a reporter vector containing a mutation in the putative miR-30d-binding site. These findings indicate that miR-30d regulates SOX4 by directly interacting with the 3′-UTR of SOX4 mRNA. We further observed an inverse expression correlation between miR-30d and SOX4 in pancreatic cancer tissues of ZZU cohort (Fig. 3h). We examined the TCGA dataset and also found a negative expression correlation between SOX4 and miR-30d in pancreatic cancer tissues (Fig. 3i). We divided patients in the TCGA PAAD into four groups according to the median mRNA expression levels of miR-30d and SOX4: miR-30d^low^&SOX4^high^, miR-30d^low^&SOX4^low^, miR-30d^high^&SOX4^high^, and miR-30d^high^&SOX4^low^. Patients in the miR-30d^high^&SOX4^low^ group showed significantly better OS (median: 71.68 months vs. 20.6 months, respectively; log-rank test, *P* < 0.05; Fig. 3j) and RFS (median: 32.42 months vs. 13.53 months, respectively; log-rank test, *P* < 0.01; Fig. 3k) than patients in the miR-30d^low^&SOX4^high^ group. These results show that the combination of miR-30d and SOX4 was more effective in predicting clinical outcome in pancreatic cancer patients than miR-30d alone.

### miR-30d suppresses the growth and invasion of PDAC by targeting SOX4 in vitro

**Fig. 4:**
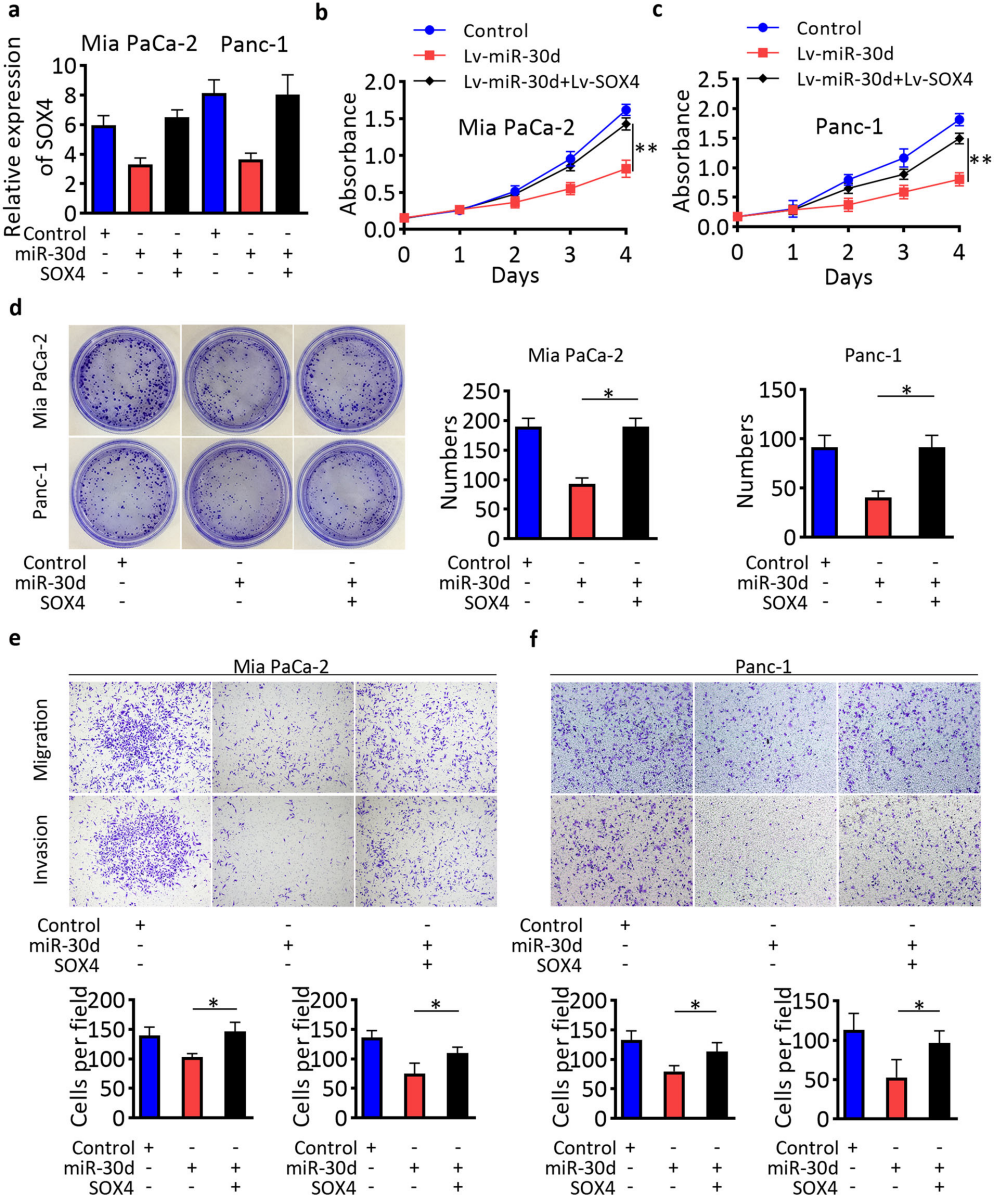
Re-expression of SOX4 attenuated the anti-cancer activities of miR-30d. **a** Expression of SOX4 in cells transfected as indicated. **b**–**d** CCK8 and colony formation assays in Mia PaCa-2 and Panc-1 cells transfected as indicated. **e**, **f** Transwell assays assessing cell migration and invasion of Mia PaCa-2 and Panc-1 cells transfected as indicated. Scale bar, 50 μm (red line). *P < 0.05, **P < 0.01, ***P < 0.001.

To explore whether the tumor suppressive effect in pancreatic cancer of miR-30d was mediated by SOX4, we first constructed miR-30d-overexpressing Mia PaCa-2 and Panc-1 cells by transfection lentivirus of Lv-miR-30d or control vectors, then overexpressed SOX4 by transfection of Lv-SOX4 lentivirus vector. The expressions of miR-30d and SOX4 were confirmed by RT-qPCR (Fig. 4a) and western blot (Supplementary Fig. S5a, b). Both CCK-8 and colony formation assays showed that while overexpression of miR-30d inhibited the proliferation abilities of pancreatic cancer cells, these effects were partially reversed after SOX4 overexpression (Fig. 4b–d). In addition, transwell assays showed that SOX4 reversed the inhibition of metastasis and invasion induced by miR-30d overexpression (Fig. 4e, f). These data confirmed that the anti-cancer activities of miR-30d in pancreatic cancer are mediated by its suppression of SOX4.

### miR-30d suppresses the SOX4/PI3K-AKT signaling pathway

**Fig. 5:**
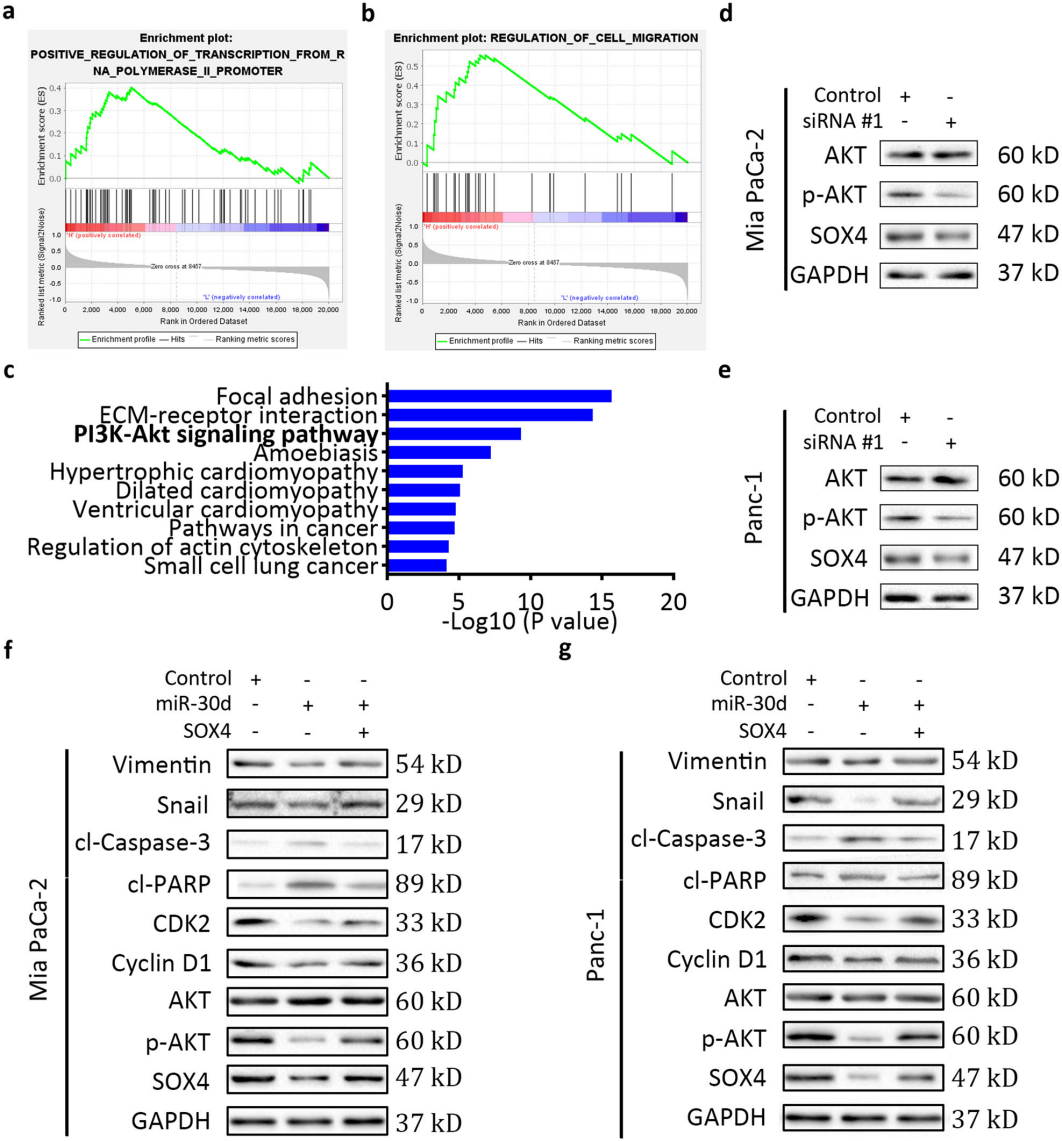
miR-30d suppresses the SOX4/PI3K-AKT-signaling pathway. **a**, **b** GSEA plots showing gene sets which are positively correlated with high SOX4 expression samples (left red) in the dataset GSE62165. **c** KEGG pathway enrichment analysis based on SOX4 expression in the TCGA dataset. **d**, **e** Western blot analysis in cells transfected as indicated. **f**, **g** Western blot analysis in cells transfected as indicated. *P < 0.05, **P < 0.01, ***P < 0.001.

To identify the mechanism of SOX4 in pancreatic cancer, gene set enrichment analysis (GSEA) with the GEO dataset GSE62165 was performed. The results revealed that gene sets associated with regulation of transcription and cell migration were significantly enriched in cases with high expression of SOX4 (Fig. 5a, b). To investigate the key signaling pathways involved in the effects of SOX4, we explored the TCGA dataset (*n* = 171) and obtained 213 genes with high correlation values (above 0.4) with SOX4, which is an activating transcription factor. The 10 most enriched signaling pathways from GO enrichment analysis are shown in Fig. 5c. We focused on the PI3K-AKT pathway, which participates in the development and progression of many cancers^22^. Knockdown of SOX4 by transfection of two independent siRNAs significantly decreased the levels of phosphorylated Akt(S473) in pancreatic cancer cells, while total Akt expression was not affected (Fig. 5d, e and Supplementary Fig. S5c, d). We next investigated whether the tumor suppressive effect of miR-30d in pancreatic cancer was mediated by the SOX4–PI3K-AKTsignaling pathway. Overexpression of miR-30d by lentivirus vector transfection reduced the levels of phosphorylated AKT(S473) in pancreatic cancer cells, which was partially counteracted by SOX4 re-expression by lentivirus vector transfection (Fig. 5f, g). Accordingly, changes of cell apoptosis, cell cycle and cell invasion regulators were also confirmed by western blotting analysis. Taken together, our results indicate that miR-30d inhibited the growth and invasion of PDAC by suppressing the SOX4–PI3K-AKTsignaling pathway.

### miR-30d/SOX4/PI3K-AKT axis suppresses the growth and invasion of PDAC in vivo

**Fig. 6:**
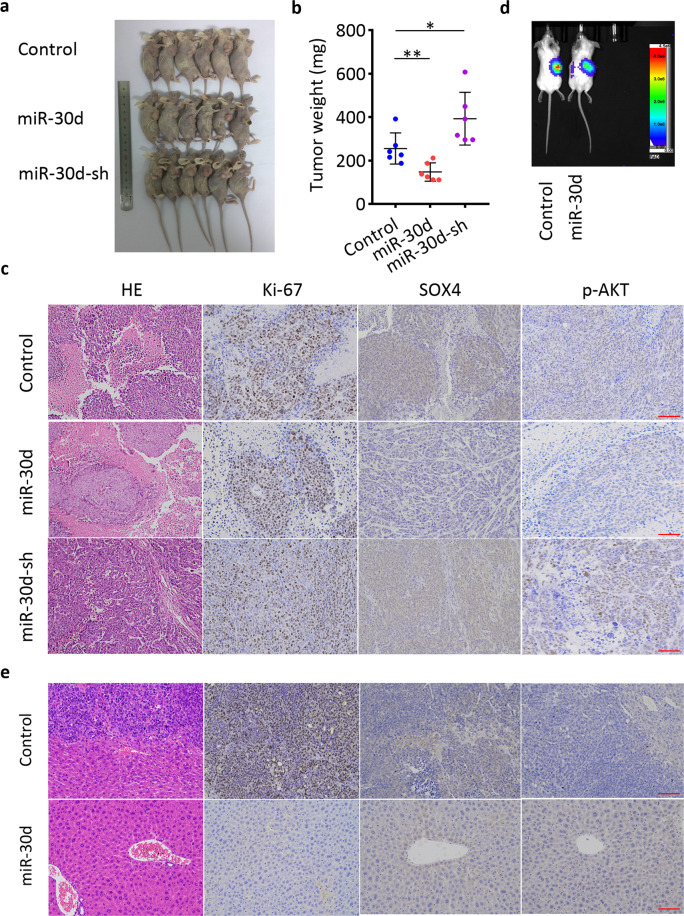
miR-30d suppresses the growth and invasion of PDAC in vivo. **a** Subcutaneous xenograft tumors in the indicated groups after stable transfection with lentivirus vectors. **b** Tumor weight of the indicated groups. **c** H&E and IHC staining of xenograft tumors of the indicated groups. Scale bar, 50 μm (red line). **d** Representative immunofluorescence images of live metastasis in the indicated groups. **e** H&E and IHC staining of nodules formed in the livers. Scale bar, 50 μm (red line). *P < 0.05, **P < 0.01, ***P < 0.001.

To validate the in vitro results of miR-30d on tumor growth, we next established a subcutaneous xenograft tumor model. Tumor weight and rate of tumor growth were significantly reduced in the miR-30d overexpression group compared with the control group (Fig. 6a, b). In contrast, tumor growth was enhanced in the miR-30d downregulated group. These observations were confirmed by hematoxylin and eosin (H&E) staining (Fig. 6c). Immunohistochemistry (IHC) analyses also showed that tumors from the miR-30d overexpression group showed reduced staining of Ki67, SOX4, and p-AKT compared with controls, while tumors from the miR-30d knockdown group showed increased staining of Ki67, SOX4, and p-AKT. To evaluate the metastatic effect of miR-30d in vitro, we then established a liver metastasis model. Fewer and smaller metastatic nodules were observed in livers from the miR-30d overexpression group compared with controls, while the miR-30d knockdown group had higher numbers and larger metastatic nodules in the liver (Fig. 6d and Supplementary Table 4). We also observed weak staining of Ki67, SOX4, and p-AKT in tumors in the miR-30d overexpression group compared with control group (Fig. 6e). Taken together, these results indicate that miR-30d/SOX4/PI3K-AKT axis suppresses the growth and invasion of pancreatic cancer cells in vivo.

### SOX4 is upregulated and associated with survival and progression in pancreatic cancer

**Fig. 7:**
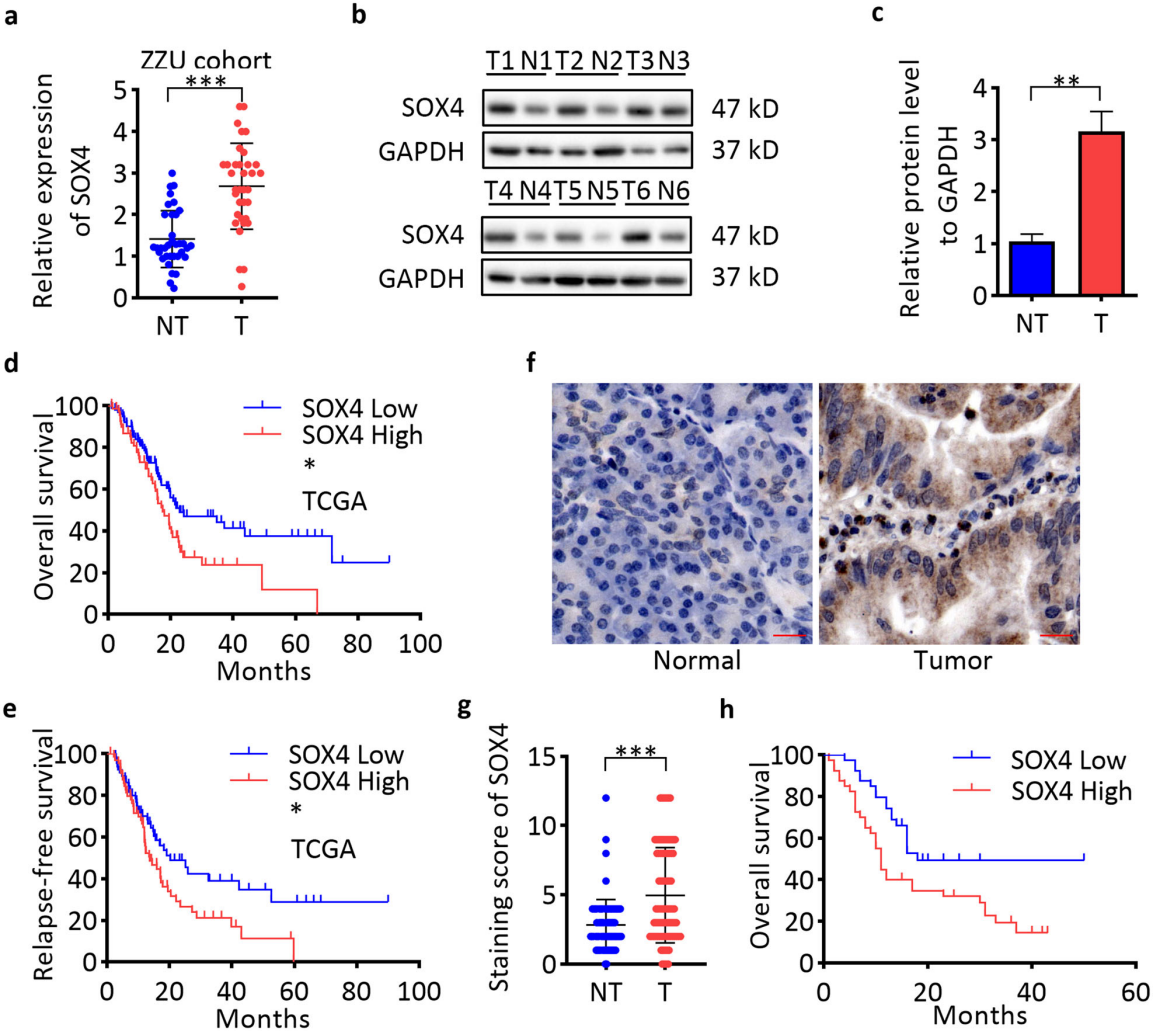
High SOX4 expression predicts the prognosis and progression of pancreatic cancer patients. **a** Detection of SOX4 mRNA expression in pancreatic cancer tissues in the ZZU cohort by RT-qPCR (*n* = 35). **b**, **c** Detection of SOX4 protein levels in pancreatic cancer tissues in the ZZU cohort by western blot (*n* = 6). **d**, **e** Kaplan–Meier analysis of the OS (*n* = 171) and RFS (*n* = 132) status of pancreatic cancer patients in TCGA based on SOX4 expression (*n* = 171). **f**, **g** IHC staining of SOX4 in pancreatic cancer tissues (*n* = 80). Scale bar, 50 μm (red line). **h** Kaplan–Meier analysis of the OS status of pancreatic cancer patients in the ZZU cohort based on SOX4 expression. *P < 0.05, **P < 0.01, ***P < 0.001.

We first mined the GEO datasets of pancreatic cancer and found that SOX4 was increased in tumor samples compared with matched non-tumor counterparts (Supplementary Fig. S6). Overexpression of SOX4 mRNA and protein in pancreatic tissues compared with non-tumor tissues was observed in RT-qPCR (Fig. 7a) and western blot assays (Fig. 7b, c). Kaplan–Meier survival analysis of patients in GSE62165 showed that pancreatic cancer patients with high SOX4 expression had worse OS compared with patients with low SOX4 expression (Supplementary Fig. S6). Analysis of the patients in the TCGA dataset also showed that patients with high SOX4 expression had worse prognosis and early relapse (Fig. 7d, e). To further explore the expression and clinical significance of SOX4 in pancreatic cancer, IHC staining was performed on in 80 pairs of PDAC tissues of ZZU cohort, and the results showed that SOX4 was significantly upregulated in PDAC tissues compared with matched non-tumor tissues (Fig. 7f, g). Correlation analysis showed that SOX4 expression was related to tumor size and tumor TNM stage (Supplementary Table 5). Survival analysis revealed that high SOX4 expression was associated with a poor clinical outcome in the ZZU cohort (Fig. 7h). IHC staining of p-AKT in pancreatic cancer tissues of the same ZZU cohort revealed a positive expression correlation between SOX4 and p-AKT (*n* = 80; Supplementary Fig. S7). These observations suggest that the miR-30d/SOX4/PI3K-AKT axis might play important roles in the tumorigenesis and metastasis of pancreatic cancer.

## Discussion

The elucidation of the molecular mechanisms underlying pancreatic cancer and identifying reliable therapeutic targets is critical in order to improve outcome of PDAC patients. Here we found that miR-30d is downregulated in pancreatic cancer and is strongly associated with PDAC progression. miR-30d inhibits pancreatic cancer cell proliferation, migration, and invasion in association with the PI3K-AKT signaling pathway by targeting SOX4. Taken together, these findings indicate that miR-30d functions as a tumor suppressor in the carcinogenesis and metastasis of pancreatic cancer.

Several studies have showed that the expression, role, and targets of miR-30d differ according to the cancer type. In non-small cell lung cancer, miR-30d is decreased and functions as a tumor suppressor by targeting CCNE2^12^. Downregulation of miR-30d promoted autophagic survival of anaplastic thyroid carcinoma cells in response to cisplatin^13^. Other studies have shown that miR-30d functions as an oncogene in different cancers. miR-30d promotes prostate cancer progression by targeting both ARID4A and ARID4B^23^. Another report showed that miR-30d promotes angiogenesis and tumor growth of prostate cancer cells via the MYPT1/c-JUN/VEGFA pathway^24^. In cervical squamous cell carcinoma, upregulation of miR-30d due to genomic amplification was associated with disease progression^25^. In our study, we found that miR-30d is decreased in pancreatic cancer and might act as a potential prognostic marker for pancreatic cancer patients. Downregulation of miR-30d was significantly correlated with a poor prognosis in PDAC. Consistent with these findings, overexpression of miR-30d significantly inhibited pancreatic cancer cell proliferation, induced G1/S cell cycle arrest and apoptosis and repressed cell migration and invasion by inducing EMT. The dysregulation of miRNAs in cancer were usually caused by genomic alteration, DNA methylation, transcription, synthesis process, nuclear export, and stability in cells. miR-30d loss was frequent in cancers, which means miR-30d might act as a vital driver gene in cancer and be a promising therapeutic target for pancreatic cancer. As K-Ras and p53 mutations are common and vital in the development and progression of PDAC, additional studies are required to elucidate the relations between them. Taken together, further studies are required to clarify the mechanisms for the loss and action mechanisms of miR-30d in PDAC.

To investigate the underlying mechanisms underlying the tumor suppressive function of miR-30d, we examined potential targets of miR-30d by bioinformatics analyses and identified SOX4 as a target gene of miR-30d in pancreatic cancer. We identified a miR-30d binding site in the 3′ UTR of SOX4. Functional assays showed that overexpression of SOX4 could partially reverse the anti-cancer effects of miR-30d. These results demonstrate that SOX4 is a direct functional target of miR-30d. The median OS of miR-30d high, SOX4 low and miR-30d^high^&SOX4^low^ groups were 23.06, 22.83, and 71.68 months, respectively. The median DFS of miR-30d high, SOX4 low, and miR-30d^high^&SOX4^low^ groups were 20.27, 20.27, and 34.42 months, respectively. Both miR-30d and SOX4 are good markers for PDAC. But a combination of miR-30d and SOX4 are better than each separate one to identify a small group of people who had the highest or the lowest risk of death or recurrence. Our findings suggest that a combination of miR-30d and SOX4 may help to identify pancreatic cancer patients who need intensive care and early intervention at early stages.

SOX4 is a member of the HMG box superfamily and participates in various development processes as a transcriptional activator^26,27^. Several studies have shown that SOX4 is upregulated and promotes the development of cancers^28^. SOX4 is increased in lung cancer due to genomic amplification and contributes to the transforming ability of lung cancer cells^29^. Multiple studies have demonstrated the critical role of dysregulation of the PI3K-AKT-signaling pathway in carcinogenesis^30,31^. However, how the PI3K-AKT pathway is regulated in cancer is largely under-characterized. Previous studies showed that amplification of SOX4 modulates PI3K-AKT signaling in breast cancer by regulating AKT phosphorylation^18^. In cholangiocarcinoma, SOX4 is overexpressed and predicts poor prognosis^32^. In cervical cancer, SOX4 was reported to promote the progression and chemotherapeutic resistance by upregulating ABCG2 transcriptionally^33^. Here we found that SOX4 activated the PI3K-AKT pathway to promote the tumorigenesis and metastasis of pancreatic cancer. However, whether SOX4 regulate the growth and invasion abilities of pancreatic cancer cells dependent or independent on PI3K-AKT, and the detailed action mechanism are still needed to be clarified.

Here we demonstrated that miR-30d inhibits pancreatic cancer cell growth and metastasis via targeting the SOX4/PI3K-AKT-signaling pathway. Our findings further show that miR-30d and SOX4 may be valuable diagnostic markers for predicting the prognosis and recurrence of pancreatic cancer patients. This newly identified miR-30d/SOX4/PI3K-AKT axis might represent new promising therapeutic targets for pancreatic cancer.

## Materials and methods

### Pancreatic cancer tissue samples

Eighty pairs of paraffin-embedded pancreatic cancer tissues and matched non-tumor tissues were obtained at The First Hospital of Zhengzhou University between February 2011 and February 2012 after informed consent was obtained. All patients were histopathologically diagnosed as adenocarcinoma. All patients received no treatments before surgery.

### Cell culture and transfections

BxPC-3, Capan-2, Mia PaCa-2, Panc-1, and SW-1990 PDAC cells were obtained from the American Type Culture Collection (ATCC, Manassas, VA, USA). The normal human pancreatic duct epithelial (HPDE) cell line was purchased from the Cell Repository of Chinese Academy of Sciences (Shanghai, China). All cell lines were authenticated by short tandem repeat DNA profiling within 3 months and cultured according to the manufacturer’s protocol as described in our previous study^3^. All cell lines were tested for mycoplasma contamination. Inhibitors and mimics of miR-30d, two independent siRNAs against SOX4 and control mimics were purchased from RiboBio (Guangzhou, China). Transient transfection of cells was performed using Lipofectamine 3000 (Invitrogen, Carlsbad, CA, USA) at a final concentration of 50 nM. Human miR-30d knockdown, miR-30d-overexpressing or SOX4-overexpressing lentiviruses and control vectors were purchased from Genechem (Shanghai, China). Lentivirus vector transfection was performed according to the manufacturer’s instructions.

### RT-qPCR

Total RNA preparation and PCR were performed as described in our previous study^3^. Total RNA was prepared from tissues and cells using TRIZOL reagent (Invitrogen). A NanoDropND-2000 spectrophotometer (NanoDropTech, Wilmington, DE, USA) was used to evaluate the concentration and quality of total RNA. cDNA was reverse-transcribed using the Prime Script™ RT reagent Kit with gDNA Eraser (Takara, Dalian, China). RT-qPCR was carried out using the SYBR Premix Ex Taq™ II kit (Takara). GAPDH and U6 were used as the internal references for mRNA and miR-30d, respectively. The 2^−ΔΔCt^ method was used to calculate relative gene expression. Primers were designed and synthesized by RiboBio and the sequences are listed in Supplementary Table 6.

### Western blot

Cells were lysed by RIPA buffer (Beyotime Biotechnology, Shanghai, China) with protease inhibitors (Roche, Indianapolis, IN, USA) and 1 mM PMSF (Beyotime Biotechnology). The BCA-200 Protein Assay kit (Pierce, Rockford, IL, USA) was used to measure protein concentration. Protein samples were separated by SDS–PAGE and transferred to polyvinylidene difluoride membranes (Millipore, Bedford, MA, USA). Membranes were blocked with 5% non-fat milk and then incubated with corresponding primary antibodies overnight at 4 °C. After washing, the membranes were incubated with corresponding secondary antibodies. The protein bands were then visualized by enhanced chemiluminescence (Boster, Wuhan, China). The primary antibodies used in our study were as follows: SOX4 (ab90696, abcam), cleaved PARP (#5625, CST), cleaved caspase-3 (#9664, CST), CDK2 (#2546, CST), Cyclin D1 (#2978, CST), Akt (#4691, CST), Phospho-Akt (Ser473) (#4060, CST), Vimentin (10366-1-AP, Proteintech), Snail (13099-1-AP, Proteintech), and GAPDH (#5174, CST).

### Flow cytometry analysis

Apoptosis and cell cycle were examined in transfected cells by flow cytometry, as previously described^3^.

### Colony formation assay

After transfection, 500 cells were seeded into 6 cm dishes (Corning, NY, USA). After culturing for 2 weeks, the cells were fixed and stained, and cell clones were counted using a light microscope (Nikon, Tokyo, Japan).

### Cell proliferation assay

The cell viability of transfected cells was measured using the Cell Counting Kit-8 (Dojindo Laboratories, Kumamoto, Japan) as previously described^3^. The absorbance of cells plated in 96-well plates was detected at 450 nm.

### IHC

IHC staining was performed on human or mouse tissues as previously described^19^. According to standard protocols, human or mice tissues were deparaffinized, rehydrated, heated for antigen-retrieval. After incubation with 10% goat serum, sections were added primary antibodies and biotinylated secondary antibodies. Staining analysis was independently performed by two pathologists. The IHC staining score was calculated as the intensity of staining multiplied by the percentage of positive cells. Staining intensity was scored as follows: 0 = negative, 1 = weak, 2 = medium, or 3 = strong. The percentage of positive cells was scored as follows: 0 = 0%, 1 = 1–25%, 2 = 26–50%, 3 = 51–75% and 4 = ≥76%. A final staining score <6 was defined as low expression and a score ≥6 was defined as high expression.

### Transwell assays

Migration and invasion assays were performed using Transwell chambers (Corning) for invasion assays, the chambers were coated with Matrigel (BD Biosciences, San Jose, CA, USA). Transfected cells were trypsinized and resuspended in 100 μL medium without serum, and the cells were placed in the top chamber. The bottom chambers contained medium with 10% fetal bovine serum. After culturing for 24 or 48 h, cells on the bottom chambers were fixed using paraformaldehyde, stained with 0.1% crystal violet and counted manually by light microscopy.

### Immunofluorescence

Cells grown on glass plates were fixed with 4% paraformaldehyde (Boster, Wuhan, China) for 15 min. Cells were permeabilized with 0.25% Triton X-100 for 10 min at room temperature and then blocked in 1% BSA in with 0.05% tween 20 for 30 min at room temperature. Cells were incubated with primary antibodies for 1 h, followed by incubation with secondary antibodies for 1 h at room temperature. Cells were counterstained with DAPI and observed and photographed using a fluorescent microscope (Nikon).

### miR-30d target gene prediction

The potential target genes of miR-30d were predicted by miRTarBase and Targetscan databases.

### Animal studies

Female BALB/c nude mice (6-weeks-old) were purchased from HFK Bioscience (Beijing, China). For the subcutaneous xenograft mouse model, 2 × 10^6^ transfected cells were injected subcutaneously into the flanks of the mice (*n* = 6 for each group) randomly. The length and width of all tumors were measured and recorded every week. After 8 weeks, mice were killed, and tumors were removed and tumor volume was calculated as (length × width^2^) × 0.5. For the liver metastasis model, Panc-1 cells were transfected with luciferase overexpressing lentivirus. Then Panc-1 cells were transfected with control vectors or miR-30d overexpressing vectors. Mice were narcotized by ether inhalation and spleens were exposed. Then 2 × 10^6^ treated Panc-1 cells (resuspended in 200 μL DMEM medium) were injected slowly into the spleen randomly. After 30 min, spleens of all mice were removed. Before taking images all mice were injected with VivoGlo luciferin (150 mg/kg, Promega, Madison, WI, USA), and image were acquired by a Bruker In-Vivo Xtreme imaging system (Bruker, Karlsruhe, Germany). Mice were killed after 8 weeks, and the livers were removed and embedded in paraffin for IHC and H&E staining. All animal experiments were approved by the Institutional Animal Care and Use Committee of The First Hospital of Zhengzhou University.

### Luciferase reporter assay

Luciferase assays were performed using the Dual-Luciferase Reporter Assay System (Promega, Madison, WI, USA) according to the manufacturer’s instructions. Pancreatic cancer cells were co-transfected with luciferase reporter vectors containing the wild-type 3′-UTR of SOX4 or the 3′-UTR in which the putative miR-30d-binding site was mutated, along with miR-30d mimics or controls. After 48 h, cells were harvested and luciferase activities were detected.

### Bioinformatic analyses

The differential expression of SOX4 mRNA between cancerous and noncancerous tissues was analyzed using public available GEO datasets by GEO2R. GSE24279 (*n* = 136), GSE60978 (*n* = 51), GSE28735 (*n* = 45), and GSE62452 (*n* = 69) datasets are surgically resected samples from PDAC patients. GSEA was performed using the GSEA software version 4.0.2 (Broad Institute, Cambridge, MA, USA)^34^.

### Statistical analyses

SPSS software 17.0 (SPSS Inc., IL, USA) and GraphPad Prism version 6.0 (GraphPad software, La Jolla, CA, USA) were used for statistical analyses. Researchers performed the measurements and statistical analysis of each group of human tissues or mice blindly. Variance is similar between the groups that are being statistically compared. *P* < 0.05 was considered statistically significant. All experiments were performed at least three times. Data are shown as mean ± SD. Chi-square (*χ*^2^) test was used to assess the correlation between the expression of miR-30d or SOX4 with clinicopathological parameters. Survival analysis was performed by the Kaplan–Meier method and Cox regression model. Expression correlation was evaluated by the Spearman correlation analysis. The differences between groups were analyzed using a paired or unpaired Student’s *t* test and one-way analysis of variance was used for analyzing differences between groups.

## Supplementary information

Supplemental Figure S1

Supplemental Figure S2

Supplemental Figure S3

Supplemental Figure S4

Supplemental Figure S5

Supplemental Figure S6

Supplemental Figure S7

Supplemental Figure S8

Supplemental table 1

Supplemental table 2

Supplemental table 3

Supplemental table 4

Supplemental table 5

Supplemental table 6

Supplementary figure legends
