# Supplementary figures and images for: miR-30d suppresses proliferation and invasiveness of pancreatic cancer by targeting the SOX4/PI3K-AKT axis and predicts poor outcome
Source: Cell Death Dis. 2021 Apr 6;12(4):350. doi: 10.1038/s41419-021-03576-0 (PMC8024348; doi:10.1038/s41419-021-03576-0)

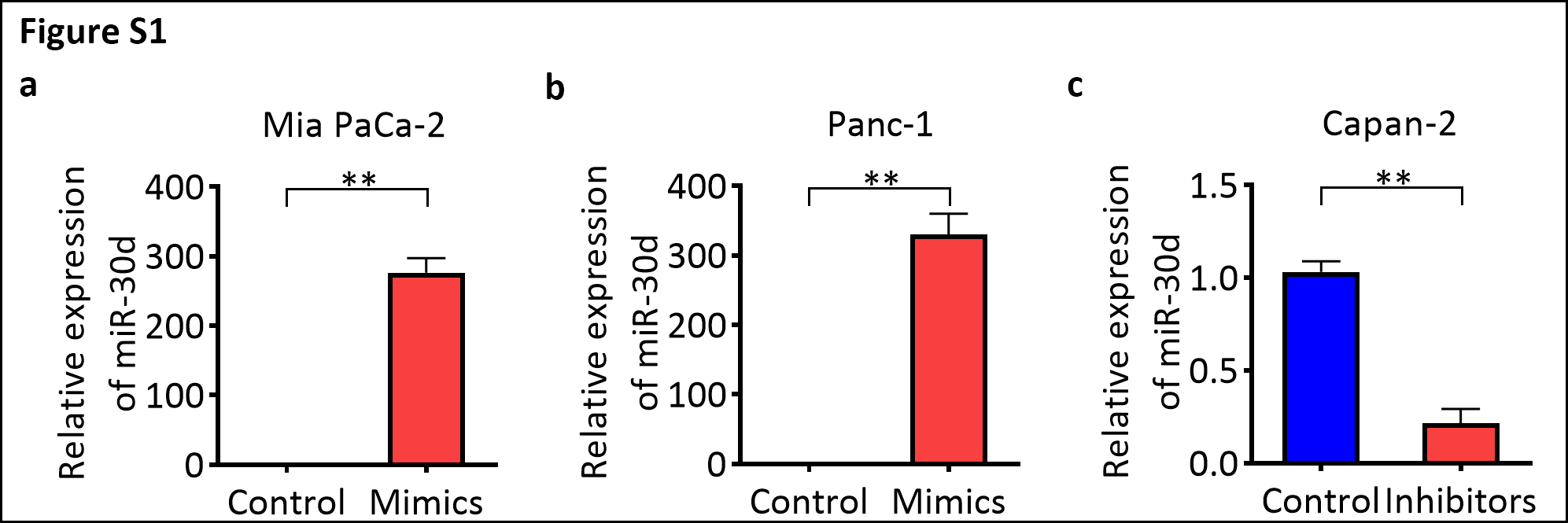

Supplement: Supplementary file 1 — Supplemental Figure S1 [file 41419_2021_3576_MOESM1_ESM.tif]

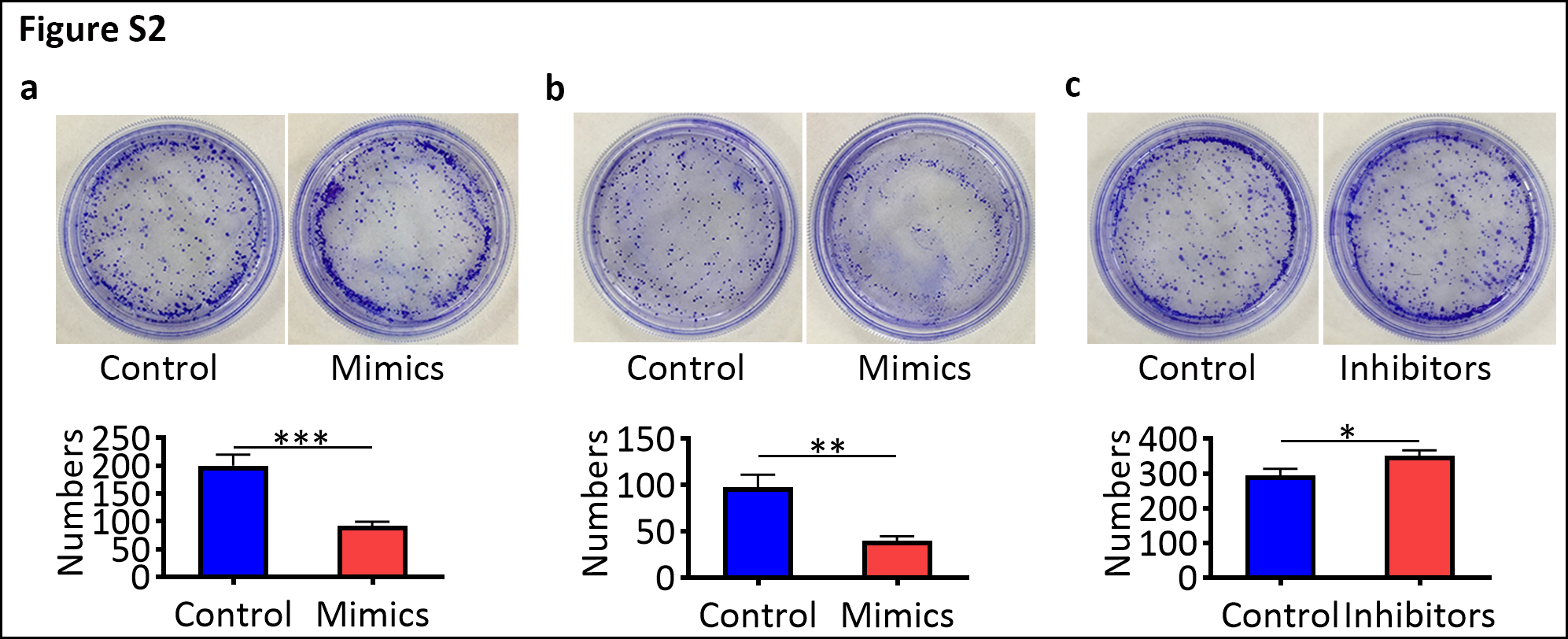

Supplement: Supplementary file 2 — Supplemental Figure S2 [file 41419_2021_3576_MOESM2_ESM.tif]

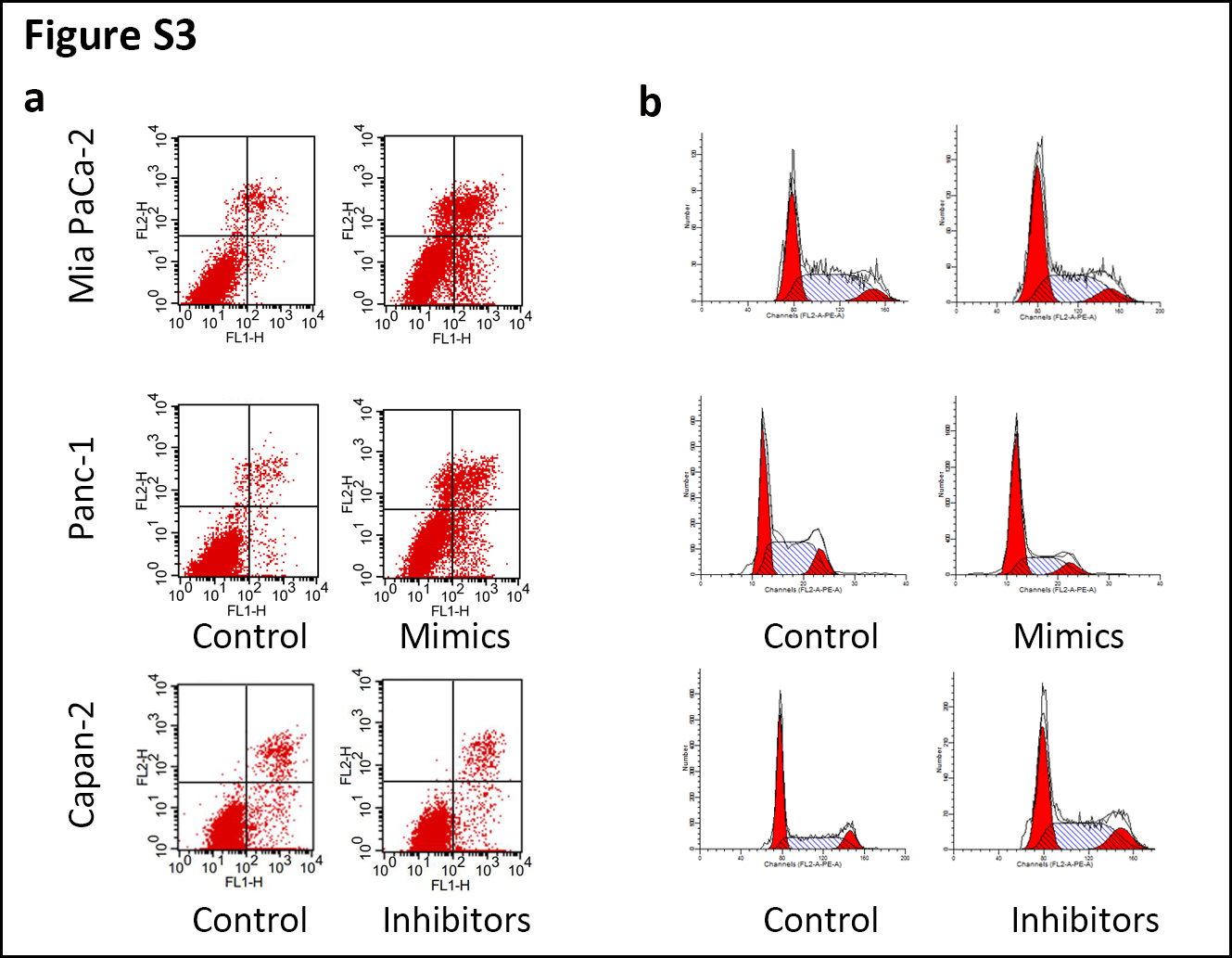

Supplement: Supplementary file 3 — Supplemental Figure S3 [file 41419_2021_3576_MOESM3_ESM.tif]

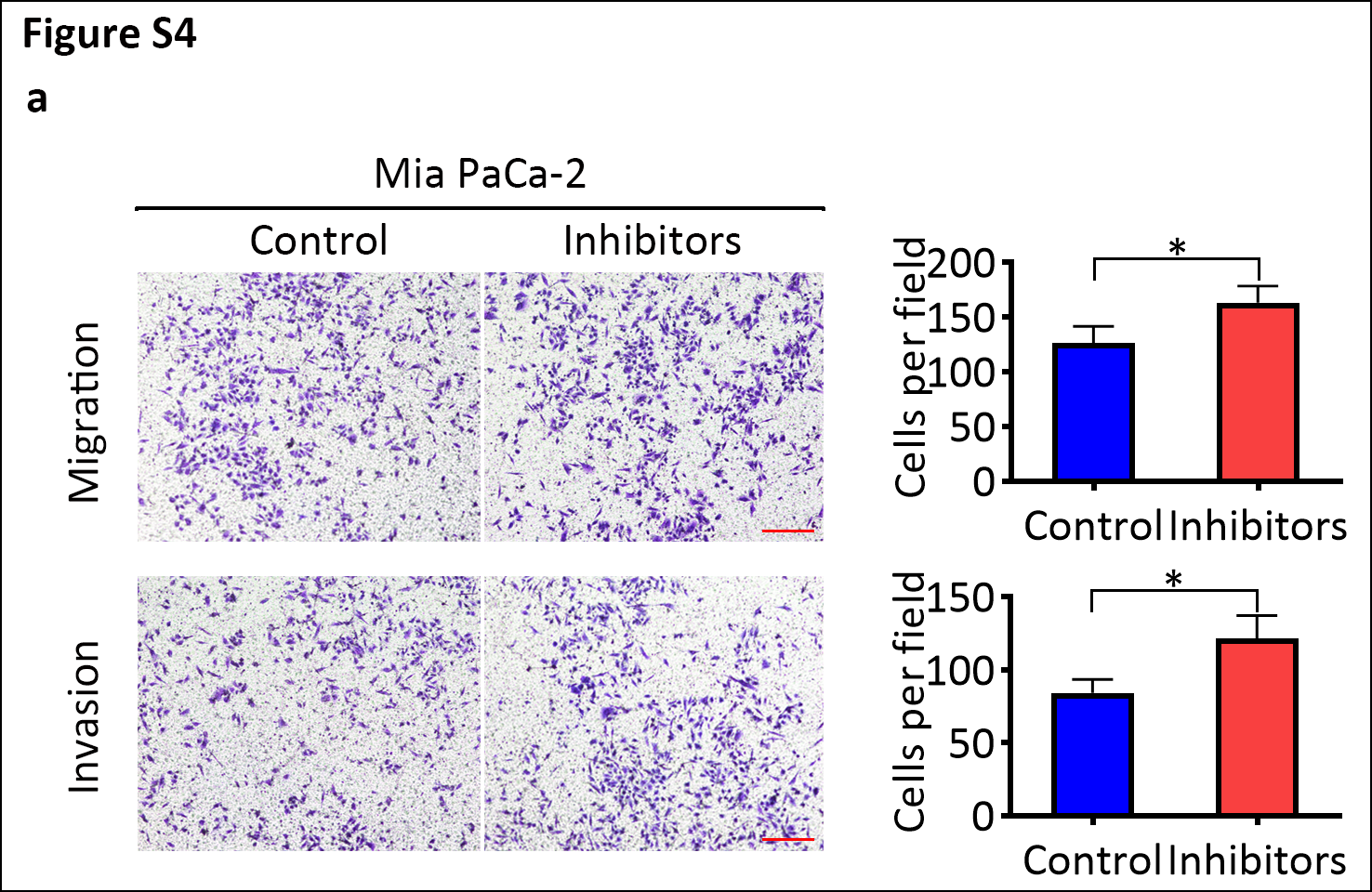

Supplement: Supplementary file 4 — Supplemental Figure S4 [file 41419_2021_3576_MOESM4_ESM.tif]

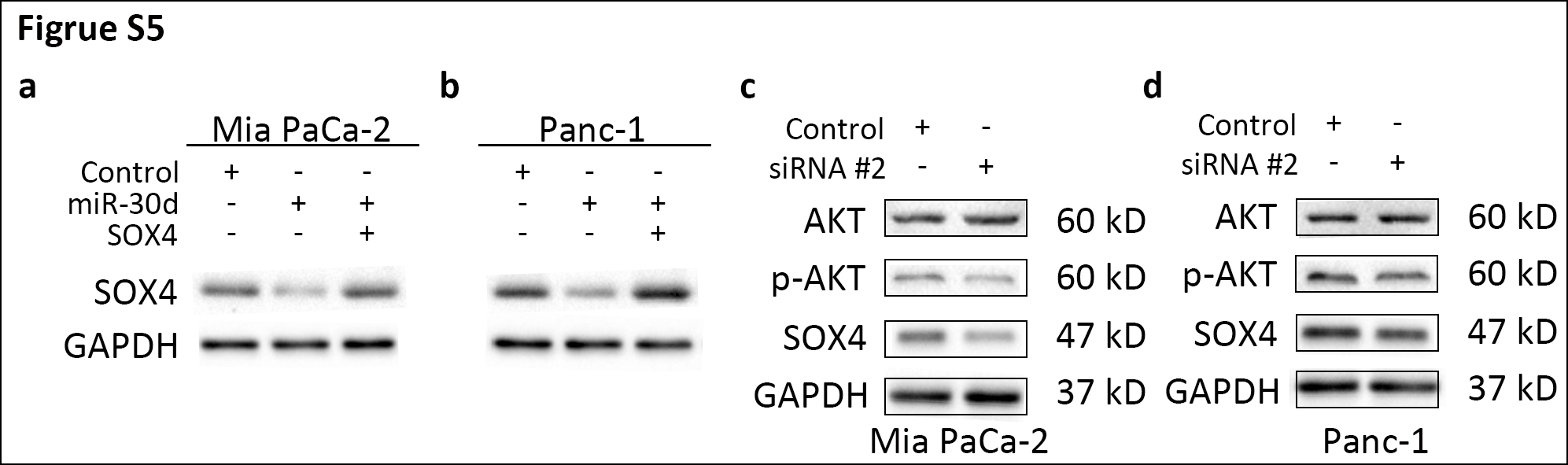

Supplement: Supplementary file 5 — Supplemental Figure S5 [file 41419_2021_3576_MOESM5_ESM.tif]

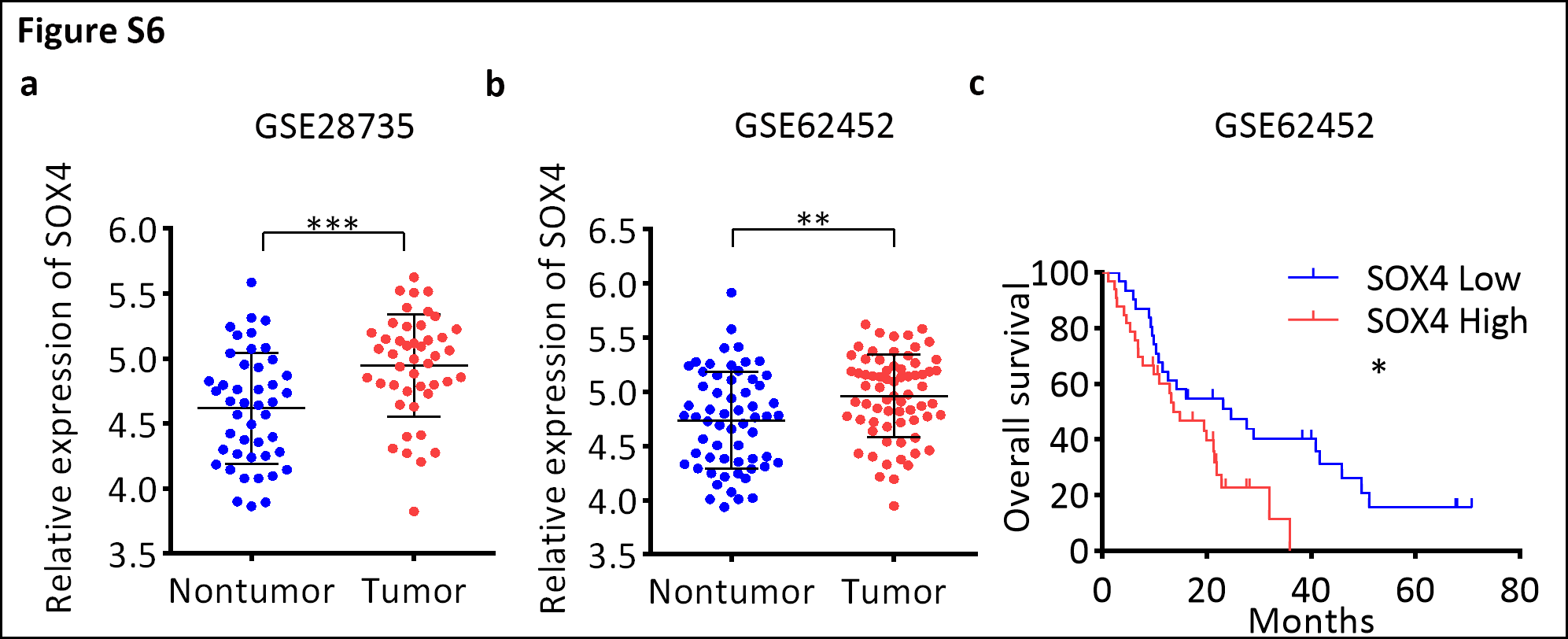

Supplement: Supplementary file 6 — Supplemental Figure S6 [file 41419_2021_3576_MOESM6_ESM.tif]

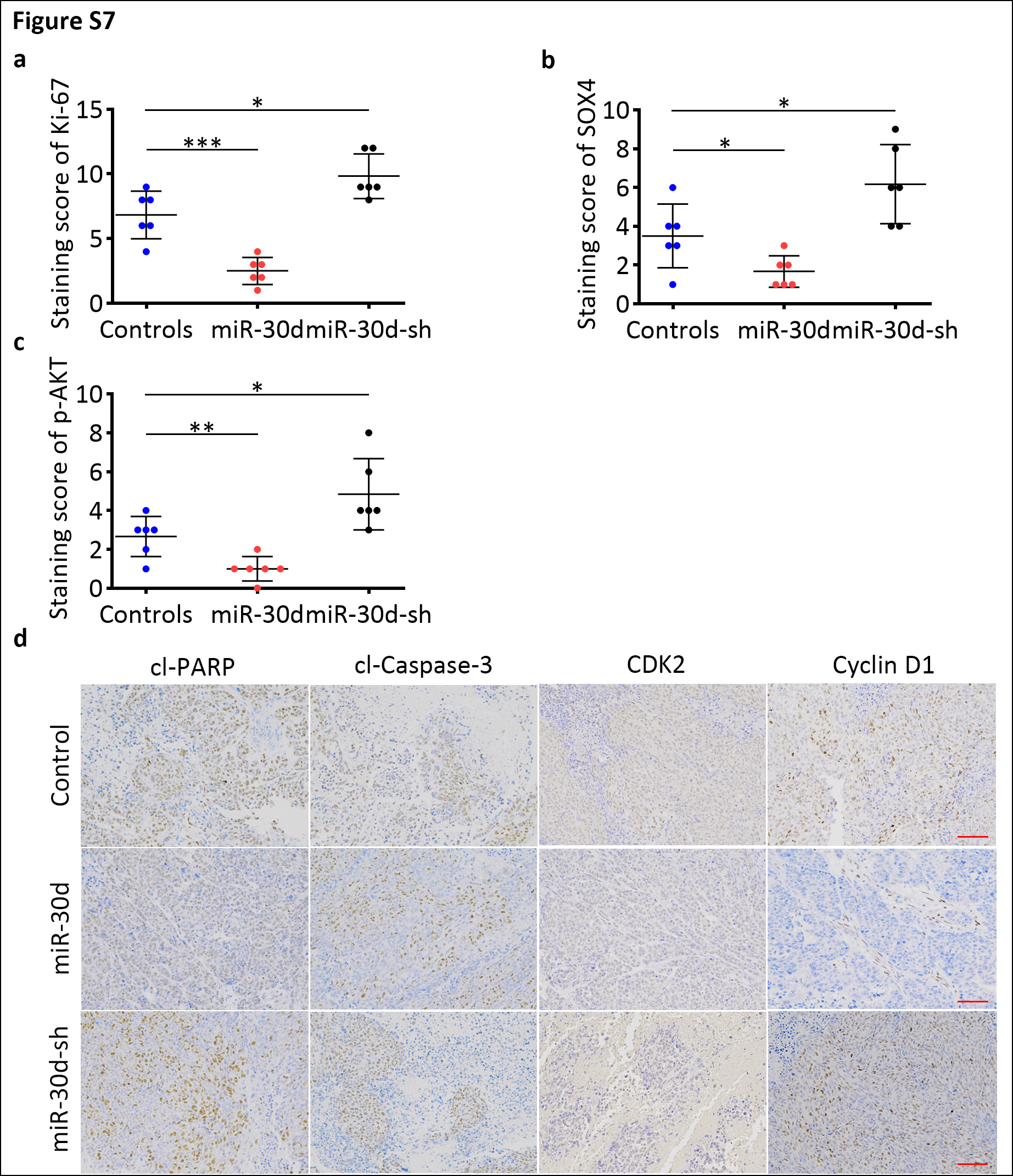

Supplement: Supplementary file 7 — Supplemental Figure S7 [file 41419_2021_3576_MOESM7_ESM.tif]

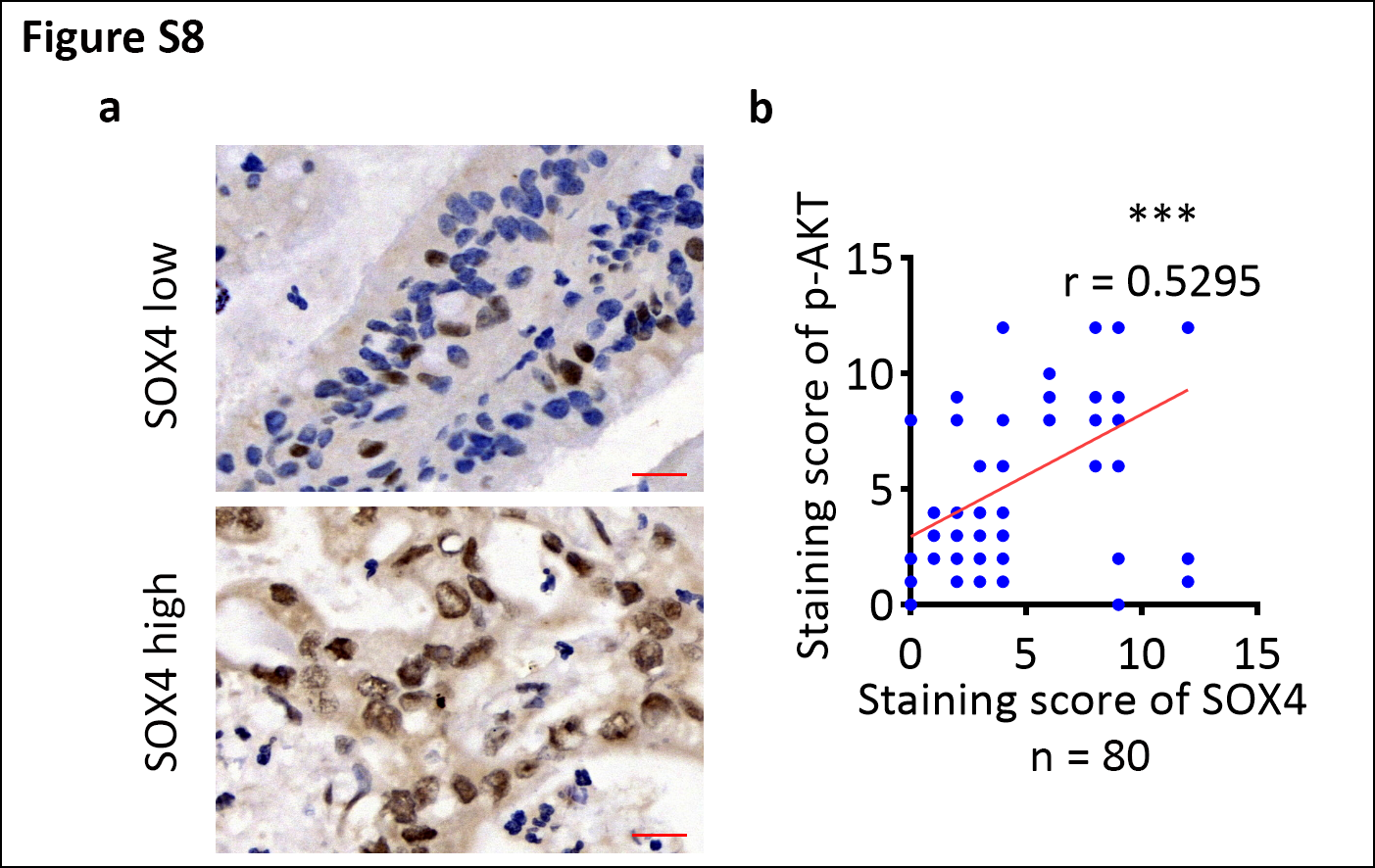

Supplement: Supplementary file 8 — Supplemental Figure S8 [file 41419_2021_3576_MOESM8_ESM.tif]
