# Supplementary material for: miR-30d suppresses proliferation and invasiveness of pancreatic cancer by targeting the SOX4/PI3K-AKT axis and predicts poor outcome
Source: Cell Death Dis. 2021 Apr 6;12(4):350. doi: 10.1038/s41419-021-03576-0 (PMC8024348; doi:10.1038/s41419-021-03576-0)
Supplement: Supplementary file 9 — Supplemental table 1 [file 41419_2021_3576_MOESM9_ESM.docx]

**Table 1** Univariable and multivariable Cox regression analysis of OS in pancreatic cancer patients in the TCGA dataset.

| Characteristic |  | Univariable analysis | | | Multivariable analysis | | |
| --- | --- | --- | --- | --- | --- | --- | --- |
|  |  | HR | 95% CI | *P*-value | HR | 95% CI | *P*-value |
| Sex | Female/Male | 1.270 | 0.669-2.410 | 0.465 | 1.310 | 0.706-2.63 | 0.389 |
| Age, years | ≥65/<65 | 1.436 | 0.739-2.792 | 0.286 | 1.526 | 0.812-2.869 | 0.186 |
| TNM | III.IV/I.II | 0.931 | 0.115-3.652 | 0.846 | 0.954 | 0.325-3.502 | 0.946 |
| Tumor grade | III.IV/I.II | 1.547 | 0.797-3.159 | 0.189 | 1.587 | 0.797-3.159 | 0.189 |
| Size, cm | ≥2.5/<2.5 | 1.454 | 0.602-3.409 | <0.01 | 1.654 | 0.896-3.105 | <0.01 |
| miR-30d | Low/High | 2.496 | 1.291-3.334 | <0.001 | 1.985 | 1.152-2.986 | <0.001 |
