# Supplementary material for: miR-30d suppresses proliferation and invasiveness of pancreatic cancer by targeting the SOX4/PI3K-AKT axis and predicts poor outcome
Source: Cell Death Dis. 2021 Apr 6;12(4):350. doi: 10.1038/s41419-021-03576-0 (PMC8024348; doi:10.1038/s41419-021-03576-0)
Supplement: Supplementary file 10 — Supplemental table 2 [file 41419_2021_3576_MOESM10_ESM.docx]

**Table 2** Univariable and multivariable Cox regression analysis of RFS in pancreatic cancer patients in the TCGA dataset.

| Characteristic |  | Univariable analysis | | | Multivariable analysis | | |
| --- | --- | --- | --- | --- | --- | --- | --- |
|  |  | HR | 95% CI | *P*-value | HR | 95% CI | *P*-value |
| Sex | Female/Male | 1.069 | 0.562-1.986 | 0.863 | 1.043 | 0.652-1.670 | 0.860 |
| Age, years | ≥65/<65 | 1.09 | 0.695-1.396 | 0.614 | 1.11 | 0.694-1.774 | 0.664 |
| TNM | III.IV/I.II | 2.231 | 1.236-2.963 | <0.05 | 2.666 | 0.914-7.778 | <0.05 |
| Tumor grade | III.IV/I.II | 1.366 | 0.987-2.398 | 0.231 | 1.462 | 0.906-2.359 | 0.119 |
| miR-30d | Low/High | 1.526 | 0.963-2.369 | <0.001 | 1.863 | 1.344-2.698 | <0.001 |
