# Supplementary material for: miR-30d suppresses proliferation and invasiveness of pancreatic cancer by targeting the SOX4/PI3K-AKT axis and predicts poor outcome
Source: Cell Death Dis. 2021 Apr 6;12(4):350. doi: 10.1038/s41419-021-03576-0 (PMC8024348; doi:10.1038/s41419-021-03576-0)
Supplement: Supplementary file 11 — Supplemental table 3 [file 41419_2021_3576_MOESM11_ESM.docx]

**Table 3** 21 predicted candidate target genes.

| GNAI2 |
| --- |
| RUNX2 |
| KIF11 |
| PSMD7 |
| SOX4 |
| FAM91A1 |
| PLEKHO2 |
| GLCE |
| PRDM1 |
| DBF4 |
| FOXA1 |
| ELOVL5 |
| ATP2A2 |
| ADAM9 |
| SLC4A7 |
| PPP1R2 |
| PPP1R12A |
| LHFPL2 |
| IL1A |
| PPP1R14C |
| MAP4K4 |
