# Supplementary material for: miR-30d suppresses proliferation and invasiveness of pancreatic cancer by targeting the SOX4/PI3K-AKT axis and predicts poor outcome
Source: Cell Death Dis. 2021 Apr 6;12(4):350. doi: 10.1038/s41419-021-03576-0 (PMC8024348; doi:10.1038/s41419-021-03576-0)
Supplement: Supplementary file 12 — Supplemental table 4 [file 41419_2021_3576_MOESM12_ESM.docx]

**Table 4 Number of mice with live metastatic nodules of indicated groups.**

| Panc-1 | Mice with metastasis |
| --- | --- |
| Control | 3/8 |
| miR-30d | 1/8 |
