# Supplementary material for: miR-30d suppresses proliferation and invasiveness of pancreatic cancer by targeting the SOX4/PI3K-AKT axis and predicts poor outcome
Source: Cell Death Dis. 2021 Apr 6;12(4):350. doi: 10.1038/s41419-021-03576-0 (PMC8024348; doi:10.1038/s41419-021-03576-0)
Supplement: Supplementary file 13 — Supplemental table 5 [file 41419_2021_3576_MOESM13_ESM.docx]

**Table 5 Correlation between SOX4 expression and clinicopathological characteristics of 80 patients of ZZU cohort.**

| Characteristics | SOX4 | | | |
| --- | --- | --- | --- | --- |
|  | All cases | Low expression | High expression | *P* value |
| Age (years) |  |  |  | 0.12 |
| ≤50 | 48 | 28 | 20 |  |
| >50 | 32 | 13 | 19 |  |
| Sex |  |  |  | 0.544 |
| Male | 45 | 25 | 20 |  |
| Female | 35 | 12 | 13 |  |
| Tumor size (cm) |  |  |  | < 0.05 |
| ≤2 | 31 | 15 | 16 |  |
| >2 | 49 | 12 | 37 |  |
| TNM stage |  |  |  |  |
| I-II | 38 | 18 | 20 | 0.083 |
| III-IV | 42 | 12 | 30 |  |
| Lymph node |  |  |  | < 0.05 |
| Negative | 25 | 18 | 7 |  |
| Positive | 55 | 24 | 31 |  |
