# Supplementary material for: miR-30d suppresses proliferation and invasiveness of pancreatic cancer by targeting the SOX4/PI3K-AKT axis and predicts poor outcome
Source: Cell Death Dis. 2021 Apr 6;12(4):350. doi: 10.1038/s41419-021-03576-0 (PMC8024348; doi:10.1038/s41419-021-03576-0)
Supplement: Supplementary file 14 — Supplemental table 6 [file 41419_2021_3576_MOESM14_ESM.docx]

**Table 6** Relative primer and siRNA sequences used in this research (5’-3’).

| GAPDH forward | CTCACCGGATGCACCAATGTT |
| --- | --- |
| GAPDH reverse | CGCGTTGCTCACAATGTTCAT |
| SOX4 forward | AGCGACAAGATCCCTTTCATTC |
| SOX4 reverse | CGTTGCCGGACTTCACCTT |
| U6 forward | CTCGCTTCGGCAGCACA |
| U6 reverse | AACGCTTCACGAATTTGCGT |
| SOX4 siRNA#1 | GCGACAAGATCCCTTTCATT |
| SOX4 siRNA#2 | GAAGAAGGTGAAGCGCGTCTA |
