# Supplementary material for: miR-30d suppresses proliferation and invasiveness of pancreatic cancer by targeting the SOX4/PI3K-AKT axis and predicts poor outcome
Source: Cell Death Dis. 2021 Apr 6;12(4):350. doi: 10.1038/s41419-021-03576-0 (PMC8024348; doi:10.1038/s41419-021-03576-0)
Supplement: Supplementary file 15 — Supplementary figure legends [file 41419_2021_3576_MOESM15_ESM.docx]

**Figure S1** **a**-**c** Expression of miR-30d after transient transfection with lipofectamine.

**Figure S2** **a**-**c** Colony formation assays of Mia PaCa-2 (left)，Panc-1 (middle) and Capan-2 (right) cells after transient transfection with Lipofectamine.

**Figure S3** **a** Cell apoptosis analysis by flow cytometry in cells after transient transfection with lipofectamine. **b** Cell cycle analysis by flow cytometry in cells after transient transfection with lipofectamine.

**Figure S4** **a** Transwell assays without or with matrigel coating to assess cell migration and invasion activities, respectively, in cells transfected as indicated. Scale bar, 50 μm (red line).

**Figure S5** **a**-**d** Western blot analysis of SOX4 expression in cells transfected as indicated.

**Figure S6** **a**-**b** Relative expression of SOX4 in GEO datasets. **c** Kaplan–Meier analysis of the OS status of pancreatic cancer patients in GSE62452 based on SOX4 expression (n = 61).

**Figure S7 a**-**c** IHC staining score of Ki-67, SOX4 and p-AKT in three groups of subcutaneous xenograft tissues (n = 6). **d** IHC staining score of cl-PARP, cl-Caspase-3, CKD2 and Cyclin D1 in three groups of subcutaneous xenograft tissues (n = 6). Scale bar, 50 μm (red line).

**Figure S8 a** IHC staining of p-AKT in pancreatic cancer tissues of ZZU cohort (n = 80). **b** Spearman correlation analysis between SOX4 and p-AKT expression by IHC in PDAC tissues. Scale bar, 50 μm (red line).
